# New York Letter

**Published:** 1896-11

**Authors:** L. B. Grandy

**Affiliations:** New York


### CORRESPONDENCE.

### NEW YORK LETTER.

New York, October 16, 1896.

Since my last letter signs of returning activity in medical and surgical circles have become more and more in evidence. The most prominent medical men have returned from their vacations and have resumed their posts in the colleges and in the clinics; medical societies are again in session, and the colleges are now in full swing.

It will be a pleasure to any one who is interested in medical teaching to visit the College of Physicians and Surgeons. Through the munificence of the Vanderbilts this institution now enjoys almost unlimited facilities, so far as wealth, apparatus, and clinical material are concerned. It is now the foremost medical school in this country. Seven years ago the college erected handsome new buildings at the corner of Fifty-ninth street and Amsterdam avenue, and within the last two years the capacity of all these buildings, including the college proper, the Vanderbilt clinic, and the Sloane Maternity Hospital, has been doubled. It is well worth one's time to spend half a day at least in these buildings to see how a modern model medical school should be conducted. The college is now about to inaugurate a thorough postgraduate course, for the instruction of practitioners in all branches of medicine and surgery. It goes without saying that it will be as complete in its way as the undergraduate school. The postgraduate department will probably be opened within a year.

Last evening I attended a meeting of the ophthalmological section of the Academy of Medicine. The general subject for discussion was the Relations of Diseases of the Eye to General Diseases. The first paper was read by Dr. J. H. Claiborne upon The Effects of Extrinsic Poisons on the Eye. In other words, Toxic Amblyopia. Following the suggestions of Dr. Casey A.

Wood, he divided these amblyopias into two general classes: (*a*) those produced by poisons which directly affect the optic nerves, and (*b*) those which are not accompanied by optic nerve or retinal lesions. In the first class the most important and frequent toxic agents which we encounter are alcohol and tobacco. It is the frequent drinker, one who drinks regularly every day, rather than the occasional heavy drinker, that is more liable to alcoholic amblyopia. With both alcohol and tobacco there is a great difference in persons as to susceptibility to these poisons. Aside from the general indications of chronic alcoholism patients with alcoholic amblyopia will complain of a misty vision and usually consult the oculist for glasses. Examination will usually show a white optic disk, especially on the temporal side. There may be some hemorrhages in the retina. Central scotoma, or color-blindness at the macula, is an early and important symptom. Tobacco amblyopia is commonest between the ages of forty and fifty. It is caused by both chewing and smoking, and it has been seen among snuff-dippers. The stronger the tobacco used the greater the liability. Dr. Claiborne had seen two cases which were caused by cigarettes, though these patients also indulged freely in alcohol. The use of tobacco and alcohol so often go hand in hand that it is not always possible to know which is responsible for the given case of amblyopia. The ocular symptoms and appearances are not sufficiently unlike for a reliable differential diagnosis to be made. In amblyopia from both poisons the first essential, of course, in treatment is total abstinence. This will effect a cure in many cases. Others will require certain drugs which will either stimulate the optic nerve or arrest the degenerative processes, such as bichloride of mercury, iodide of potash, strychnine, digitalis, pilocarpine, etc. The author mentioned among the other less important drugs of this class which may cause amblyopia by optic nerve lesions, cannabis indica, arsenic, lead, quinine, iodoform, salicylic acid, male fern, iodide of potassium, etc.

The other class of poisons produces amblyopia without any retinal or optic nerve lesions. These are: (1) agents which dilate the pupil, such as belladonna or atropine, cocaine, hematropine, hyoscyamine, scopolamine, etc.; (2) those which contract the pupil, such as opium, eserine, pilocarpine, etc.; and (3) those which produce irregular visual disturbances, as santonin, aniline, naphthaline, picric acid, ergot, etc.

In concluding his excellent paper Dr. Claiborne stated that the possible visual effects of such drugs as may cause amblyopia should be well borne in mind by the practitioner.

Upon the discussion of this paper Dr. Herman Knapp called attention to the fact that persons with alcoholic or tobacco amblyopia become prematurely presbyopic. In his experience tobacco amblyopics had always begun the use of tobacco early in life, before twenty. Persons beginning the tobacco habit later in life are not so liable to it. Dr. Knapp reported a case of amblyopia from coal-gas.

Other interesting papers were read by Dr. C. J. Kipp, on The Infectious Diseases of the Eye, and Dr. W. A. Holden, on The Visual Disturbances due to Nervous Diseases.

At the Brooklyn Eye and Ear Hospital a few days ago I witnessed an operation for an old middle-ear and mastoid suppuration, which presented more than the usual points of interest. The patient's trouble dated from surf-bathing, July 4th. This produced an acute otitis, accompanied by pain; paracentesis was performed by some aurist, and a discharge continued for three weeks. For the next two months there was no further discharge, only pain in the affected half of the head, and finally vertigo, occasional vomiting and increasing weakness. The physician who was attending the patient at this time did not seem to realize the gravity of the case. When he came to the hospital, September 23d, there was no trace of mastoid tenderness, no pus in the canal, bnt marked drooping of the postero-superior canal wall. Vision and eye-grounds normal, and for twenty-four hours after pulse and temperature were normal. Then symptoms of meningitis appeared, patient became delirious, temperature 102, pulse weak and rapid, 120. The attending surgeon, Dr. J. Sheppard, decided to operate at once. The mastoid cells were opened and found to contain a considerable quantity of pus, granulations, and necrosed bone. As these were not considered sufficient to account for the grave cerebral symptoms, it was decided to explore the temporal lobe of the brain for abscess. The skull was trephined about one inch above the auditory canal, and the dura mater was found congested and inflamed. The temporal lobe of the brain was punctured by the trocar in several directions, but no pus could be obtained. In the next forty-eight hours the patient's condition showed much improvement; meningitic symptoms disappeared. There was suppression of urine on second day, and a uremic convulsion. Urine found to be heavily loaded with albumin. Other convulsions followed, and patient died on the third day. It was Dr. Sheppard's opinion that operation should have been done earlier, before the development of the meningitis. The symptoms exhibited by the patient for two months before he came to the hospital would have amply justified an exploration of the mastoid.

For the benefit of practitioners who wish to come here to study eye and ear and throat diseases, I will mention the following institutions which should be visited: The New York Eye and Ear Infirmary, 13th street and 2d avenue; Manhattan Eye and Ear Hospital, 41st street and Park avenue; New York Ophthalmic and Aural Institute (Dr. Knapp), 46th East 12th street; Metropolitan Throat Hospital, 351 West 34th street; Eye and Ear and Throat Departments of the Vanderbilt Clinic, 60th street and Amsterdam avenue; and the New Amsterdam Eye, Ear, and Throat Hospital, 212 West 38th street. There are others, but these are the most important. These departments in the Polyclinic and the Postgraduate should of course be mentioned. Dispensaries and Out-Patient Departments of the larger hospitals, of which there are a great many, always have a large attendance of eye, ear, and throat patients, and one who has access to these places can use them to great advantage. The attending staff at many of these institutions are always willing to accept assistance, and those who express a desire to get some practical clinical work will usually have little difficulty in finding it.

L. B. GRANDY, M.D.

